# Correlation Between Cerebral Venous-Arterial Pco_2_ Difference With Jugular Venous Oxygen Saturation As Global Cerebral Ischemia in Severe Traumatic Brain Injury

**DOI:** 10.1186/2197-425X-3-S1-A492

**Published:** 2015-10-01

**Authors:** E Gómez-Sandoval, M Hernandez-Flores, R Soriano-Orozco, MN Gómez-Gonzalez, MDR Valdez-Medina

**Affiliations:** Universidad de Guanajuato, Medicine, Guanajuato, Mexico

## Introduction

Traumatic Brain Injury (TBI) remains a leading cause of morbidity and disability and is considered a major public health concern. Ischemia is considered a relevant factor in TBI for prognosis, but its diagnosis in the early phases is still a problem. Now jugular venous oxygen saturation (SjVO_2_) is used to evaluate blood flow and cerebral metabolic rate as well as the cerebral venous-arterial pCO2 difference (VADpCO_2_) as a marker of low cerebral blood flow. Besides, there's no statement of VADpCO_2_ to guide medical therapy, consider as a not specific for global cerebral ischemia, but its observation over time may be useful.

## Objectives

The goal of this study was to compare the correlation between VADpCO_2_ and SjVO_2_ on the assessment global cerebral ischemia in severe TBI.

## Methods

This prospective observational, correlational study was conducted at the Intensive Care Unit Centro Médico Nacional del Bajío UMAE 1, in León Guanajuato, between August 2013 and December 2014. Patients were admitted in the intensive care unit after severe TBI, defined by an admission Glasgow Coma Scale < 8. We placed a catheter on right jugular bulb in all patients and corroborate the localization by X-Ray and CT-Scan. We took serial samples of jugular bulb catheter and arterial blood gases every 6 hours from admission or if the patient had a neurological worsening. the measurements of the SjVO_2_ were assessment in normal flow between 55-75%, high cerebral blood flow > 75% and low cerebral blood flow < 55%. the data were recorded such a VADpCO_2_ in each measurement.

## Results

**Figure 1:**
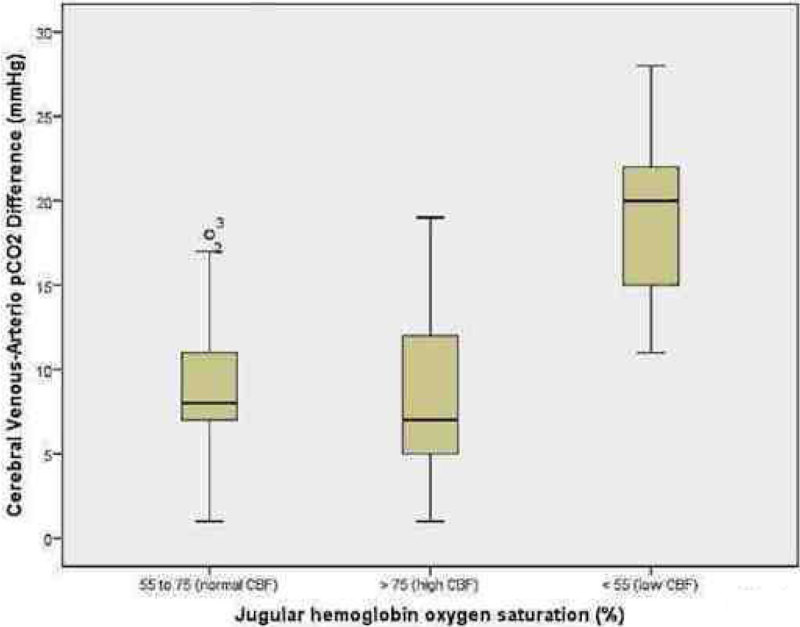
***VADpCO***_***2***_ ***and cerebral blood flow.***

**Figure 2:**
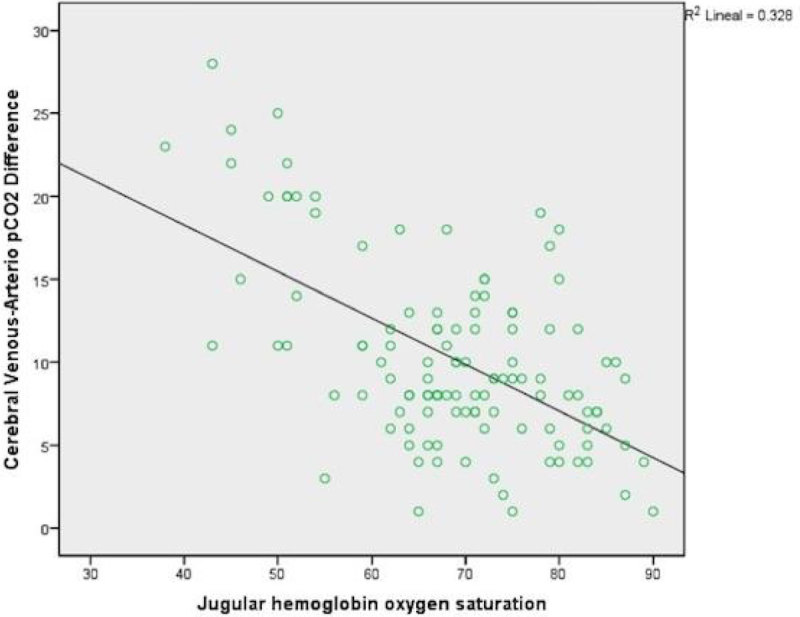
***Correlation between VADpCO***_***2***_ ***and SjO***_***2***_

We included twenty-nine patients in the study, with a median age of 36 (interquartile range 24-56) years). One hundred and sixteen samples were taken. the distribution was 14.6% for low cerebral flow, 53.4% for normal cerebral flow and 31.8% for high cerebral flow. There was a statistically significant difference between the groups with VADpCO_2_ F (_2,113_)= 45.29, p= < 0.001. There was a change in the median of VADpCO_2_ in the different subgroups, SjVO_2_ < 55% (19.12 ± 5.08 mmHg), SjVO_2_ 55 to 75% (8.94 ± 3.63mmHg) and SjVO_2_> 75% (8.3 ± 4.58 mmHg), with and alpha error of 0.05 (Figure 1), and moderately strong negative correlation between SjVO_2_ y VADpCO_2_ r=- 0.57 (Figure 2)

## Conclusions

There is a moderately strong correlation between SjVO_2_ and VADpCO_2_ that may be used for the correlation for global cerebral ischemia in the assessment of multimodality monitoring in neurocritical care in severe TBI.

## Grant Acknowledgment

This study did not receive any grant from any funding agency.
